# A Phosphorus–Nitrogen Synergistic Flame Retardant for Enhanced Fire Safety of Polybutadiene

**DOI:** 10.3390/polym18010127

**Published:** 2025-12-31

**Authors:** Hongwu Zhang, Huafeng Wei, Heng Yue, Mingdong Yu

**Affiliations:** 1 Shandong Electric Power Industry Boiler and Pressure Vessel Inspection Center Co., Ltd., Jinan 250002, China; 2 State Grid Shandong Electric Power Research Institute, Jinan 250003, China; 3 Department of Materials Science and Engineering, University of Jinan, Jinan 250022, China

**Keywords:** flame retardancy, polybutadiene, DOPO derivative, high efficiency

## Abstract

Polybutadiene has excellent mechanical properties and flexibility. It is widely used in elastomers and industrial fields. However, it has the characteristic of high flammability. The low LOI and rapid heat release upon ignition pose significant fire hazards. This results in a significant fire safety risk during service. Therefore, its application in some key fields has been restricted. In this study, polybutadiene with high-performance flame-retardant properties was developed by adding phosphorus–nitrogen synergistic flame retardants to address this challenge. This flame retardant mainly enhances its flame retardancy through the synergistic gas-phase and condensed-phase mechanisms. Dense and continuous carbon layers could be promoted by flame retardants during combustion. It provides an effective thermal barrier and oxygen barrier. In addition, phosphorus-containing volatiles can function by suppressing flame propagation via radical quenching in the gas phase. The modified polybutadiene reached UL-94 V-1 grade at the optimal load of 1.0 wt%. Meanwhile, its LOI increased to 27%. The cone calorimeter test further confirms a high reduction in peak heat release rate (pHRR). This work provides a feasible strategy for developing advanced polybutadiene materials. It can effectively enhance its fire safety. At the same time, it maintains a balance between flame retardancy and the overall material performance.

## 1. Introduction

Polybutadiene (PBD) is widely used in construction and transportation because of its elasticity, processability, and designable formulations. However, the hydrocarbon-rich backbone makes PBD highly flammable. Its limiting oxygen index (LOI) is usually below 18% [1,2,3]. During combustion, PBD can exhibit high heat release and intense smoke production. This kind of hazard poses a serious threat to life and property safety. It also significantly limits its application in applications with stringent fire-safety requirements [4,5,6]. Therefore, improving the flame retardancy of PBD has become important, driven by stricter safety regulations and growing demand for fire-safe materials [7,8,9,10,11,12,13]. Various strategies have been explored in recent years. Among them, phosphorus–nitrogen (P-N) synergistic systems are widely used to improve fire performance while retaining mechanical integrity [14,15,16,17].

Various flame-retardant systems have been explored to reduce the fire risk of PBD. These approaches mainly include halogenated, phosphorus-based, and inorganic flame retardants [18,19,20,21]. However, halogenated flame retardants may release corrosive/toxic gases during burning and raise environmental concerns [22,23,24]. Therefore, they are being progressively restricted or phased out [25,26,27]. Moreover, increasing evidence has revealed complex photolytic and metabolic transformation pathways for legacy and emerging brominated flame retardants, suggesting that substitution decisions should be evaluated from a full life-cycle perspective [28,29,30,31]. Inorganic flame retardants often require high loadings, which can impair processability and mechanical strength [7,8]. Therefore, efficient and environmentally benign halogen-free systems are of strong interest [9,10,11,12,13,21]. Among the candidate systems, Phosphorus-based flame retardants attract interest because they can act in both the gas and condensed phases and are effective at relatively low loadings [14,15,16,17].

Against this background, 9, 10-dihydrogen-9-oxygen-10-phosphazafine-10-oxide (DOPO) and its derivatives are regarded as ideal halogen-free substitutes [15,16,17]. This type of compound has high P-H bond reactivity and good thermal stability, and can form a dense and stable carbon layer at the same time. DOPO functions both by scavenging reactive radicals in the gas phase and by facilitating the generation of a protective char layer in the condensed phase, thereby achieving effective combustion suppression. In addition, when phosphorus-containing compounds are used in combination with silicon-containing or nitrogen-containing additives, their flame-retardant performance can be further enhanced [21,25,26,27].

Previous studies have explored the flame retardancy and performance balance of the PBD system from different perspectives. Mishra et al. [32] used PBD rubber as the matrix and systematically compared the reinforcing and flame-retardant effects of nano CaCO_3_ and fly ash fillers. The results showed that nano CaCO_3_ could significantly enhance the mechanical properties under the same filling conditions. The fillers also had an impact on the combustion behavior. This demonstrated the “strengthening–flame retardancy” synergistic effect of inorganic/nano fillers in the PBD system. Liu et al. [33] constructed an expansion-type flame retardant system based on APP. The result revealed that pyrolysis and combustion process could be regulated by the synergistic effect of metal oxides. Amrollahi et al. [34] studied the influence of HBCD on HTPB-based PBD adhesives. They pointed out that the improvement in flame retardancy is often accompanied by a sacrifice in mechanical/adhesive properties or thermal stability. This illustrates the contradiction in the “flame retardant efficiency–mechanical retention” aspect within the PBD system. In addition to the additive route, Kim et al. [35] modified the PBD and polyurethane composite system by introducing structural design strategies such as POSS. As a result, better ablation resistance and flame retardancy were achieved. This indicates that macroscopic structure/network design is an effective method to enhance combustion safety. Recently, Wiśniewska et al. [36] summarized and modeled a large amount of data on flame-retardant rubber composites. The overall patterns and trade-off relationships of different flame-retardant systems in terms of LOI, UL-94, and cone calorimetry indicators have been summarized. This also provides a direction for the design of flame retardants for rubber materials.

However, halogen-free flame-retardant PBD still faces a pronounced trade-off between heat-release suppression and increased smoke generation, and DOPO-derived systems in PBD have not been systematically investigated. Herein, a Bis-DOPO Schiff-base is introduced to construct a P–N synergistic PBD system. The novelty lies in a systematic structure–property–fire performance correlation (mechanical/thermal properties and LOI/UL-94/cone calorimetry) that quantifies this trade-off and identifies an optimal loading for the best overall balance [14,15,16,17,28,29,30,31,32,37]. The Bis-DOPO Schiff-base was prepared according to the reported two-step procedure, involving Schiff-base formation (DDM with 4-hydroxybenzaldehyde) and subsequent P–H addition of DOPO to the imine (C=N) groups [37]. This structure exhibits a significant phosphorus-nitrogen synergistic effect and shows high application potential in the field of polymer flame retardancy. In this study, the Bis-DOPO Schiff-base formula reported in the literature was used to modify PBD. The composite materials with different amounts were prepared. The samples were named PBD-0.5, PBD-1, and PBD-1.5 in sequence. This work conducted a systematic study through Fourier Transform Infrared Spectroscopy (FTIR), thermogravimetric analysis (TGA), mechanical property testing, limiting oxygen index (LOI), vertical burning (UL-94), cone calorimetry, scanning electron microscopy (SEM), and energy dispersive spectroscopy (EDS) to evaluate the thermal stability, mechanical behavior, and flame retardancy of materials. These tests aim to clarify their flame-retardant mechanism and provide a basis for the optimal design of phosphorus-containing flame retardants in the modification of PBD.

## 2. Experimental Section

### 2.1. Materials

4,4′-Diaminodiphenylmethane (DDM), p-hydroxybenzaldehyde, 9,10-dihydro-9-oxa-10-phosphaphenanthrene-10-oxide (DOPO) were purchased from Shanghai Macklin Biochemical Technology Co., Ltd. (Shanghai, China). Absolute ethanol was supplied by Sinopharm Chemical Reagent Co., Ltd. (Beijing, China). Resin-grade PBD (Mn = 3700 g/mol; -NCO content 2.5%; 40 °C viscosity 15 Pa·s) and curing agent were purchased from Jingshou New Materials Technology Co., Ltd. (Jinan, China).

### 2.2. Preparation of Flame Retardant Bis-DOPO Schiff-Base

The flame-retardant Bis-DOPO Schiff-base was synthesized according to the literature procedure reported by Xu et al. [37]. Briefly, 4,4′-diaminodiphenylmethane (DDM, 1.98 g, 0.01 mol) and 4-hydroxybenzaldehyde (2.44 g, 0.02 mol) were dissolved in 1,4-dioxane (40 mL) in a three-necked flask under a nitrogen atmosphere. The mixture was stirred at 55 °C for 2 h to form the bis-Schiff-base intermediate. Subsequently, a 1,4-dioxane solution (20 mL) containing DOPO (4.32 g, 0.02 mol) was added dropwise, and the reaction was continued at 55 °C for 12 h to afford the Bis-DOPO Schiff-base structure (Scheme S1 in the Supporting Information). After cooling to room temperature, the reaction mixture was poured into ice water, and the precipitate was collected by filtration, washed twice with ethanol, and vacuum-dried at 70 °C for 24 h to obtain a light-yellow powder.

The reported yield in the reference is 92.4%, and the melting point determined by DSC is 157.0 °C. In this work, the successful formation of the intermediate and the final product was further verified by FTIR and XPS analyses. The corresponding spectra/thermograms are provided in the Supporting Information (Figures S1 and S2). The obtained spectral features are consistent with those reported in the reference.

### 2.3. Preparation of Flame-Retardant Modified PBD

#### 2.3.1. Preparation of PBD for the Blank Group

Blank samples of resin-grade PBD-0 were prepared by the solution casting method. First, PBD resin was dissolved in toluene. The mixture was magnetically stirred at room temperature for 4 h until a uniform and transparent PBD solution was obtained. The curing agent was added and thoroughly mixed. Subsequently, the solution was placed under vacuum conditions (≈−0.08 MPa) for deaeration for 15 min to remove the mixed air bubbles. The deaerated solution was slowly poured into the pre-prepared standard plate mold. The solvent was slowly evaporated in a forced-convection drying oven at 60 °C for 12 h to form a film and allow the sample to set. The material was further vacuum-dried at 60 °C for 8 h to completely remove residual solvent. After cooling to room temperature, the sheet was carefully demolded to obtain a smooth, light-yellow PBD blank specimen, which was denoted as PBD-0. The PBD-0 samples were subsequently used for mechanical testing, thermal analysis, and flame retardancy evaluation.

#### 2.3.2. Preparation of Flame-Retardant Modified PBD

PBD resin was weighed according to the formulations listed in Table 1. Toluene was subsequently added to an appropriate amount to obtain a solid content of 15–20 wt%. The mixture was magnetically stirred at room temperature for 4 h until PBD was completely dissolved, resulting in a uniform and transparent PBD solution. Subsequently, the predetermined mass of Bis-DOPO Schiff-base flame retardant was weighed (Table 1) to add to the solution. The flame retardant was ultrasonically dispersed at 60 °C for 2 h. The above-mentioned Bis-DOPO solution was slowly poured into the PBD toluene solution. The mixture was further stirred at room temperature for 2 h to obtain a macroscopically homogeneous blend. The curing agent was added and thoroughly mixed. Then the solution was degassed under vacuum (≈−0.08 MPa) for 15 min to remove trapped bubbles. The deaerated solution was slowly poured into the pre-prepared standard plate mold. The solvent is gradually evaporated in a forced convection drying oven at 60 °C for 12 h. Then it is transferred to a vacuum drying oven and further vacuum-dried at 60 °C for 8 h to fully remove the residual solvent. After cooling to room temperature, the sheets were carefully demolded to obtain flame-retardant PBD specimens with uniform thickness and a smooth surface, denoted as PBD-0.5, PBD-1, and PBD-1.5.

### 2.4. Material Characterization

The chemical structure of the material was analyzed using a Nicolet iS10 Fourier Transform Infrared (FTIR) Spectrometer (Thermo Fisher Scientific, Madison, WI, USA) with a scanning range of 4000–500 cm^−1^. The thermal stability of the Polybutadiene material was characterized using a TGA55 thermogravimetric analyzer (Waters, New Castle, DE, USA). The test was conducted under an air atmosphere with a heating rate of 10 °C/min, ranging from 50 to 800 °C, using a sample mass of 5 mg. Tensile and flexural strength were measured using a universal testing machine (WDW-100E, Jinan Kason Testing Equipment Co., Ltd., Jinan, China) according to GB/T 1040 and GB/T 9341, respectively. The tests were conducted at room temperature under a constant crosshead speed. At least five specimens were tested for each formulation. The apparent density of the PBD was calculated according to Equation (1), following the GB/T 6343-2009 standard. Samples with dimensions of 50 mm × 50 mm × 50 mm were accurately weighed using an analytical balance with a precision of 0.001 g.

(1) $$\rho =\frac{m}{v}\times 1000$$

*m*: Mass of the specimen, in grams (g);

*v*: Volume of the specimen, in cubic centimeters (cm^3^);

*ρ*: Apparent density of the specimen, in kilograms per cubic meter (kg/m^3^).

The limiting oxygen index (LOI) test was conducted according to the GB/T 2408-2021 standard method using an LOI tester (JF-5, Cangzhou Jingwei Instrument Equipment Manufacturing Co., Ltd., Cangzhou, China) on the prepared Polybutadiene materials. The sample dimensions were 80 mm × 10 mm × 4 mm. Ten samples were tested for each group, and the average value was calculated from the test results. The vertical burning test was performed with reference to the GB/T 2408-2021 method using a UL-94 tester (TTech-GBT2408, Testech (Suzhou) Testing Instrument Technology Co., Ltd., Suzhou, China) on the prepared Polybutadiene materials. The sample dimensions were 125 mm × 13 mm ×3 mm. The cone calorimetry test was carried out according to the ISO 5660-1 standard, with a heat flux of 50 kW/m^2^. The standard sample dimensions were 100 mm × 100 mm × 3 mm. All quantitative results are reported as mean values (n ≥ 5 for mechanical tests and n = 10 for LOI). Statistical analysis data are reported as mean ± SD with the number of replicates (n). One-way ANOVA was used to evaluate the significance of differences between PBD-1 and PBD-1.5. A *p* value < 0.05 was considered statistically significant. The microscopic morphology of the PBD material and the residual char after the cone calorimetry test was characterized using a QUANTA 250 FEG scanning electron microscope (FEI, Hillsboro, OR, USA). The samples were sputter-coated with a thin layer of gold prior to characterization. The elemental composition of the PBD material and the residual char after the cone calorimetry test was characterized using a QUANTA 250 FEG scanning electron microscope. The elemental composition and chemical states of the residual char from the flame-retardant PBD material after the cone calorimetry test were characterized using an ESCALAB Xi+ X-ray photoelectron spectrometer (Thermo Scientific, East Grinstead, UK).

## 3. Testing and Analysis Section

### 3.1. Fundamental Testing

**Figure 1:**
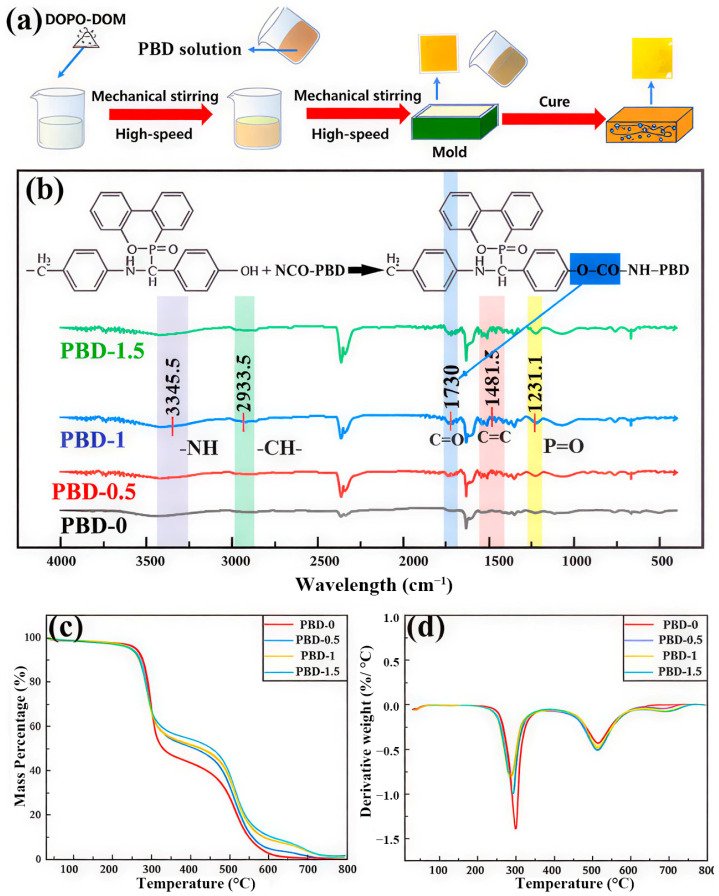
(**a**) Diagram illustrating the preparation process of flame-retardant PBD; (**b**) FT-IR spectra of PBD-0, PBD-0.5, PBD-1, and PBD-1.5; (**c**) TG and (**d**) DTG curves of PBD-0, PBD-0.5, PBD-1, and PBD-1.5.

The synthesis of Bis-DOPO Schiff-base is illustrated in Figure 1a. The FT-IR results (Figure 1b) verify that the flame-retardant component has been effectively integrated into the PBD system. In addition, Figure 1b provides a comparative overview of the IR spectra for PBD-0, PBD-0.5, PBD-1, and PBD-1.5. A new absorption peak was observed at 1231.1 cm^−1^ in the sample after adding Bis-DOPO Schiff-base compared with the unmodified PBD-0, which did not exist in the blank sample and could be attributed to the P=O stretching vibration of the phosphorus-containing moiety. This characteristic peak gradually intensified as the flame-retardant content increased from 0.5 wt% to 1.5 wt%. This indicates that Bis-DOPO Schiff-base has been successfully introduced and dispersed relatively uniformly in the PBD matrix. The content of available phosphorus in the system monotonically increases as the proportion continues to rise. A pronounced intensification of the absorption band in the modified sample was observed near 1481.5 cm^−1^. This can be attributed to the stretching vibration of the aromatic C=C backbone in Bis-DOPO. This indicates that as flame retardants are introduced, the proportion of rigid aromatic structures in the system increases. This will have a certain hardening effect on the movement of PBD chain segments and the local microstructure. The bandwidth at 2933.5 cm^−1^ mainly corresponds to the non-symmetric stretching band of the –CH_2_ group. The minor elevation in peak strength can be ascribed to the combined contribution of the PBD main chain and the methylene group in the flame-retardant molecule. This indicates that the introduction of flame retardants did not damage the main structure of PBD but was embedded in the matrix in a blending manner. Furthermore, a wide and weak absorption band appeared in the modified samples at approximately 3345.5 cm^−1^, while it was basically not obvious in PBD-0. This band can be attributed to the N–H stretching vibration in the Bis-DOPO Schiff-base molecule. Notably, the appearance of the N–H stretching band at ~3345.5 cm^−1^ together with the carbonyl band at ~1730 cm^−1^ (C=O) is consistent with the formation of urethane (–O–CO–NH–) linkages. This suggests that Bis-DOPO Schiff-base is incorporated not only through physical blending but also through possible chemical interactions with the PBD network, potentially involving reactions between its phenolic –OH groups and terminal –NCO groups of NCO–PBD. Covalent coupling improves interfacial compatibility and helps retain P-containing moieties in the condensed phase. It is conducive to forming a more stable carbon layer during the combustion process. Its intensity progressively rises as more flame retardants are incorporated. This means that the increase in N–H content in the system is consistent with the dosage of flame retardants. Meanwhile, the absorption band is slightly broadened, indicating that there may be a certain degree of hydrogen bonding or dipole–dipole interaction between N–H and P=O or trace polar groups in the system. This enhances the intermolecular forces and, to a certain extent, restricts the free movement of PBD segments. This is mutually corroborated by the subsequent changing trends of heat resistance and mechanical performance.

The thermogravimetric analysis results (Figure 1c,d and Table 2) indicate that both the initial degradation temperature (T_5_%) and the temperature at the maximum weight-loss rate (Tmax) showed a progressive decrease with the introduction of the Bis-DOPO Schiff-base flame retardant. The carbon residue rate significantly increased at 800 °C. Specifically, the T_5_% of PBD-0 was 260.4 °C, and the Tmax decreased from 350 °C to 322.6 °C. When the flame-retardant content increased to 1.5 wt% (PBD-1.5), the T_5_% decreased to 243.9 °C. This phenomenon is consistent with the relatively low thermal stability of Bis-DOPO Schiff-base itself. Flame retardants preferentially decompose at lower temperatures, generating phosphoric acid-containing species. This plays a catalytic role in the thermal cracking of the PBD main chain, slightly advancing the initial decomposition temperature of the overall system. Meanwhile, the carbon residue rate of PBD-0 was only 0.4 wt%, while in the samples containing flame retardants, the carbon residue rate gradually increased to 3.5 wt% (PBD-1.5). Although the absolute value is still relatively low, for the PBD matrix that is almost non-carbonized by itself, this increase has already indicated that flame retardants can significantly promote the carbonization process at high temperatures. This initially indicates that the phosphorus-containing intermediate products generated by the decomposition of flame retardants promote the transformation of PBD into a carbon-rich structure through catalytic dehydration, aromatization promotion, and other pathways, thereby forming a more stable carbon layer. In addition, a secondary degradation peak can be observed at higher temperatures in the DTG curves (Figure 1d), which is typical for tests conducted under an air atmosphere. This second peak is attributed to the further oxidative decomposition/consumption of the preformed carbonaceous residue generated in the first stage, rather than additional scission of the original PBD backbone. Notably, the flame-retardant samples show a less pronounced and slightly broadened high-temperature peak, suggesting that the phosphorus-containing residue improves the oxidation resistance of the carbonaceous layer, consistent with the increased residue at 800 °C (Table 2). It should be noted that the Tmax values reported in Table 2 correspond to the first DTG peak, and the maximum weight loss rate varies relatively little with the dosage of flame retardant. It slightly decreased at medium contents (PBD-0.5, PBD-1). However, it slightly increased in the high-content sample PBD-1.5. This slight fluctuation may be related to the speed and uniformity of carbon layer formation. Moderate flame-retardant content is conducive to the formation of a relatively dense and uniform surface carbon layer during the decomposition process. This slows down the rapid thermal cracking of the matrix to a certain extent. When the content of flame retardants is further increased, the decline in local compatibility and the inhomogeneity of microstructure may lead to concentrated decomposition in some areas within a narrower temperature range. This causes the peak of the DTG curve to rise slightly.

### 3.2. Mechanical Properties and Apparent Density Testing

**Figure 2:**
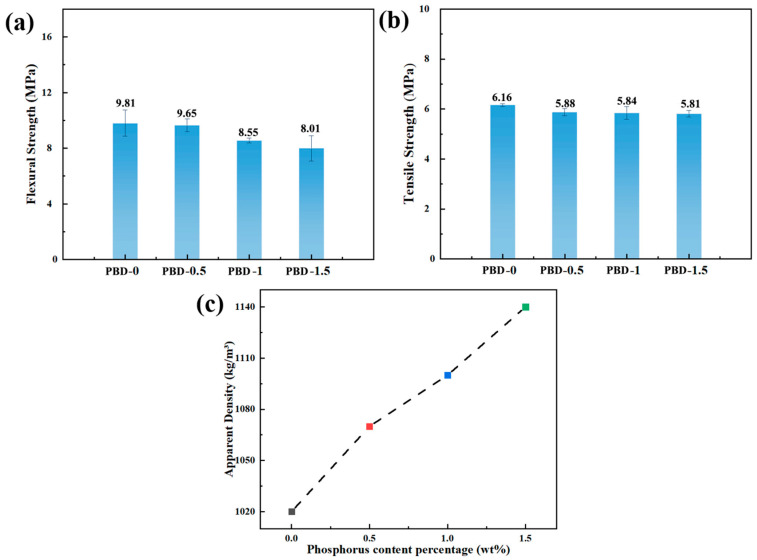
(**a**) Flexural strength, (**b**) tensile strength, and (**c**) apparent density of the flame-retardant PBD.

Figure 2a,b indicates that the bending strength of the material continuously decreases as the content of Bis-DOPO Schiff-base increases. The bending strength of PBD-0 is 9.81 MPa, while that of PBD-0.5, PBD-1, and PBD-1.5 drops to 9.65, 8.55, and 8.01 MPa, respectively. The flame retardant contains a rigid aromatic structure. Its polarity and volume are significantly higher than those of the PBD segments. Therefore, it is difficult to achieve complete uniform dispersion in the matrix. Local enrichment regions are prone to forming stress concentration points under bending load. This causes the bearing path of the continuous segments to be disrupted and microcracks to be induced, thereby leading to a more significant decrease in bending strength. The change in tensile strength is relatively small. The tensile strength of PBD-0 is 6.16 MPa, while that of PBD-0.5, PBD-1, and PBD-1.5 drops to 5.88, 5.84, and 5.81 MPa, respectively. Uniaxial tensile testing mainly relies on the continuous PBD segments to bear the load. The influence of the flame retardant on the orientation of the segments and the overall elongation behavior is limited. Therefore, the tensile strength only shows a slight attenuation. This result indicates that the sensitivity of the material to the microcompatibility differences under tensile deformation is significantly lower than that under bending load; thus, the decrease in bending strength is more prominent. Figure 2c shows that the apparent density of the material steadily increases with the increase in the content of the flame retardant. It rises from approximately 1.02 g·cm^−3^ of PBD-0 to 1.14 g·cm^−3^ of PBD-1.5. The phosphorus, nitrogen, and aromatic structure in the flame retardant have a higher mass density, and their addition directly increases the overall density of the material. At the same time, there is a certain molecular force between the flame retardant and the PBD segments. During the curing process, it can reduce free volume and improve structural density. The increase in density indicates that the rigidity of the system has increased, which is consistent with the downward trend of the bending performance. The addition of the flame retardant significantly improves the flame-retardant performance of the material while also causing certain mechanical performance losses. However, within the range of 1.5 wt%, it still maintains acceptable structural stability. The material exhibits a tunable structure–flame-retardant performance balance.

### 3.3. Vertical Burning Test and Limited Oxygen Index (LOI) Test

**Figure 3:**
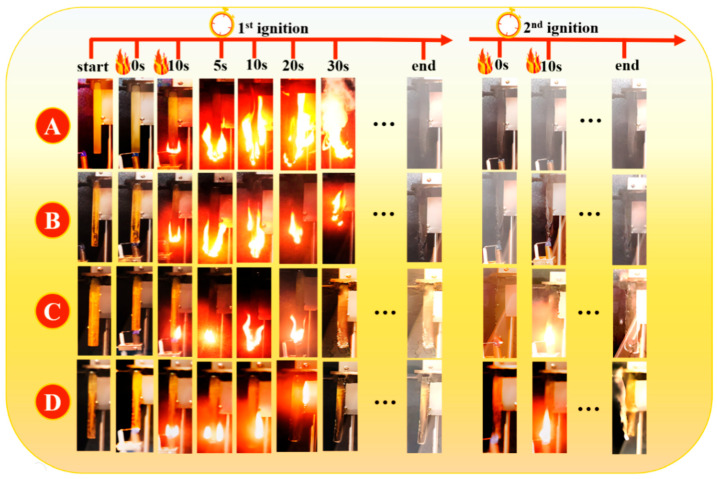
Still images from the UL-94 test video of Bis-DOPO Schiff-base flame-retardant PBD samples: (**A**) PBD-0; (**B**) PBD-0.5; (**C**) PBD-1; (**D**) PBD-1.5.

**Figure 4:**
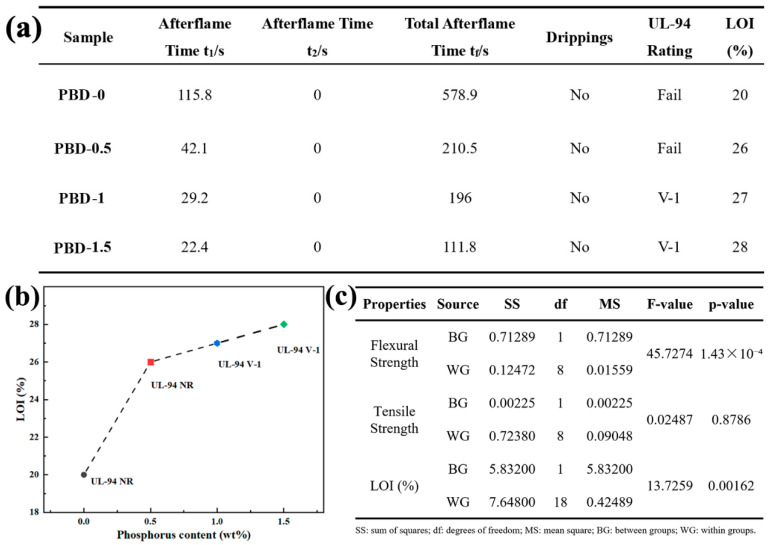
(**a**) Vertical burning test data of the flame-retardant PBD (LOI values are means ± SD, n = 10; rounded in panel (**a**) for clarity); (**b**) LOI and UL-94 ratings of PBD with different phosphorus contents; (**c**) One-way ANOVA summary for mechanical properties and LOI (PBD-1 vs. PBD-1.5).

As can be seen from the UL-94 vertical burning test and LOI results (Figure 3 and Figure 4), the flame retardancy of the material improved markedly with increasing phosphorus content. PBD-1 (1 wt% P) achieved the V-1 grade in the UL-94 test. The cumulative self-extinguishing time was significantly shortened. Meanwhile, the LOI increased to approximately 27%. This indicates that the material’s tendency to burn in an oxygen-rich environment decreases. The cumulative self-extinguishing time was further shortened to approximately 111.8 s, and the LOI increased to approximately 28% when the flame-retardant content was further increased to 1.5 wt% (PBD-1.5). This shows that the system promotes a more coherent P-containing residue, providing an auxiliary barrier effect. Although the flame-retardant performance continued to improve, the sample failed to reach the V-0 grade. This may be related to the fact that the carbon layer was not sufficiently continuous and dense in the early combustion stage. Excessively high flame-retardant content may also reduce the compatibility of the matrix, thereby slowing down the rapid formation of the protective layer. One-way ANOVA shows that increasing the loading from 1.0 to 1.5 wt% significantly improves LOI (*p* = 0.00162). Meanwhile, the flexural strength decreases significantly (*p* = 1.43 × 10^−4^), whereas tensile strength shows no significant change (*p* = 0.8786). This demonstrates that a higher flame-retardant loading improves oxygen-index-based flame resistance but significantly decreases flexural strength, highlighting the dosage-dependent trade-off in this system. The statistical results are summarized in Figure 4c. Overall, this system shows a better balance of flame-retardant structural performance by around 1 wt%.

### 3.4. Cone Calorimeter Test

**Figure 5:**
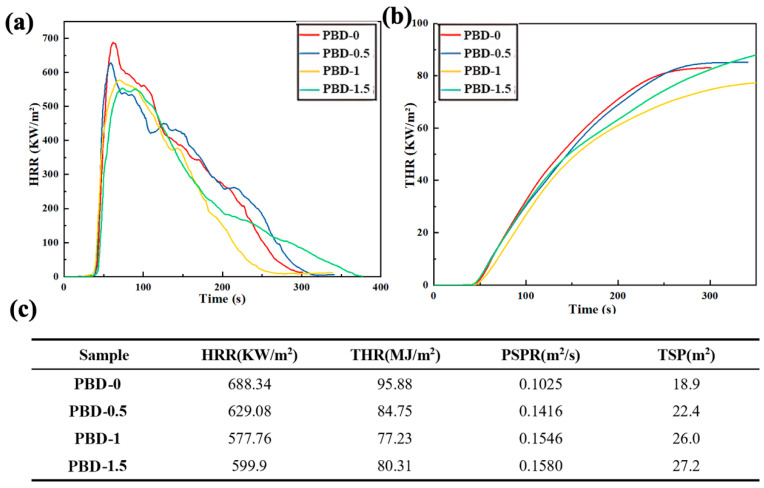
(**a**) HRR and (**b**) THR curves of Bis-DOPO Schiff-base flame-retardant PBD; (**c**) Cone calorimetry test data of the flame-retardant modified Polybutadiene.

Figure 5 shows that both the maximum heat release rate (pHRR) and the overall heat output (THR) of the material significantly decreased with the addition of Bis-DOPO Schiff-base flame retardant. Compared with the unmodified PBD-0, the pHRR of PBD-1 decreased from 688.34 kW/m^2^ to 577.76 kW/m^2^, and the THR decreased from 95.88 MJ/m^2^ to 77.23 MJ/m^2^, respectively, reducing by 16.1% and 19.6%. This indicates that the flame retardant forms a phosphorus-containing carbonized structure in the early combustion stage. This effectively weakens the flame propagation intensity and reduces the release of combustible gases. The pHRR and THR slightly increase compared to PBD-1 when the flame-retardant content is further increased to 1.5 wt% (PBD-1.5). This suggests that an excessively high content may lead to a decrease in the uniformity of the dispersion of the matrix-incorporated flame retardant, thereby affecting the density and continuity of the carbon layer and slightly reducing the fire suppression efficiency. Nevertheless, the heat release level of PBD-1.5 is still significantly better than that of PBD-0. This indicates that the interfacial interaction of the flame retardant still dominates the overall process. Along with the reduced heat release, the peak smoke production rate (PSPR) and the total smoke production (TSP, time-integrated smoke release) increased by 54% and 44%, respectively. This “lower heat release but higher smoke” behavior is frequently reported for DOPO-type phosphorus flame retardants due to their combined gas-/condensed-phase actions [38,39,40]. Phosphorus-derived species can promote dehydration/aromatization of polymer chains and facilitate the formation of a thicker protective char layer, which limits heat/oxygen transfer and slows flame oxidation [38,39]. Meanwhile, PO/PO_2_ radicals can scavenge H/OH in the flame zone, reducing combustion efficiency and leading to more incomplete oxidation of pyrolysis products, thereby increasing smoke generation [38,39]. Overall, the flame retardant effectively suppresses the heat release of PBD by enhancing condensed-phase charring, at the expense of higher smoke intensity. Considering the balance between heat-release inhibition and smoke control, PBD-1 exhibits the best comprehensive performance and is therefore regarded as the optimal loading level for this system.

### 3.5. Char Residue Testing

**Figure 6:**
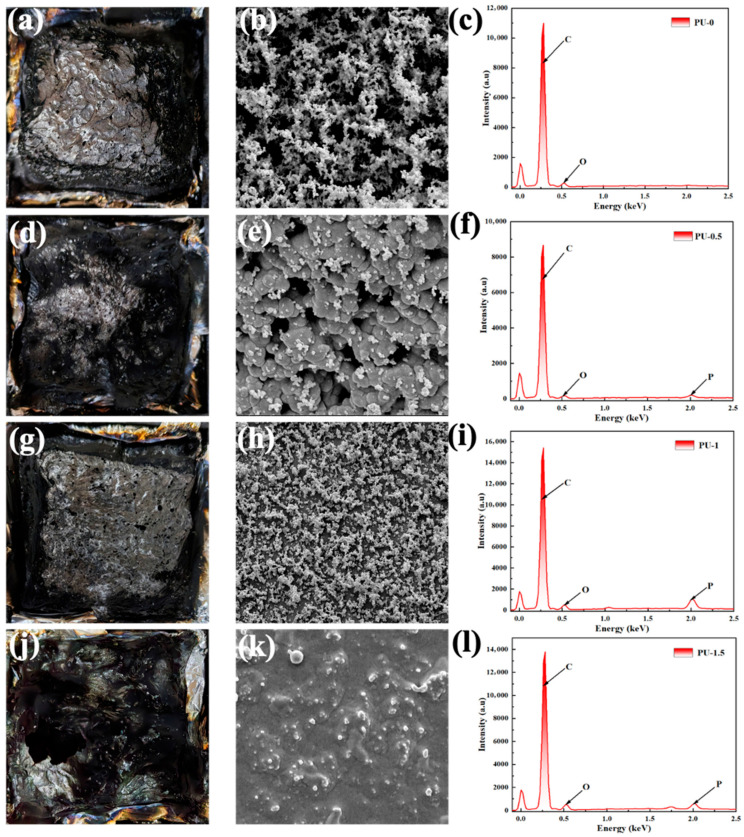
(**a**–**c**) correspond to the PBD-0 group (without flame retardant): (**a**) macroscopic appearance after combustion, (**b**) SEM micrograph showing the microscopic morphology, and (**c**) EDS spectrum; (**d**–**f**) are for the PBD-0.5 group: (**d**) macroscopic appearance, (**e**) SEM micrograph, and (**f**) EDS spectrum; (**g**–**i**) are for the PBD-1 group: (**g**) macroscopic appearance, (**h**) SEM micrograph, and (**i**) EDS spectrum; (**j**–**l**) are for the PBD-1.5 group: (**j**) macroscopic appearance, (**k**) SEM micrograph, and (**l**) EDS spectrum.

Figure 6 shows the macroscopic morphology, microstructure, and elemental composition of the residues after combustion. It should be noted that the char yield measured by TGA at 800 °C in air is relatively low (2.3–3.5%, Figure 1c), suggesting that the condensed-phase contribution is supportive rather than dominant. Accordingly, the residue morphology and elemental composition (SEM/EDS), together with the established gas-phase inhibition pathway of DOPO-type systems, are used to interpret the flame-retardant behavior [38,39,40]. The residue of neat PBD is loose and discontinuous, with a porous skeleton and thin pore walls, indicating that only a limited and fragile residue is formed during burning. The residue becomes progressively more coherent after adding the flame retardant. PBD-0.5 shows improved continuity with reduced pore size and more pronounced particulate features, suggesting the onset of a thin protective residue on the surface. PBD-1 produces the most intact and compact residue, which consists of closely stacked fine particles with fewer defective regions. This kind of morphology is expected to provide an auxiliary barrier that may hinder heat/oxygen transfer and slow the release of flammable volatiles. PBD-1.5 still shows a relatively dense residue. However, local bulges and irregular shrinkage are observed. This suggests that excessive loading may induce micro-heterogeneity and reduce residue uniformity.

EDS analysis shows that the char residue of neat PBD contains only C and O, whereas the flame-retardant samples exhibit a distinct P signal. The increased phosphorus signal with increasing loading indicates a higher retention of phosphorus-containing species in the residue. Such P enrichment is generally associated with enhanced residue integrity and oxidation resistance, thereby contributing to a supportive condensed-phase protection [38,39,40].

**Figure 7:**
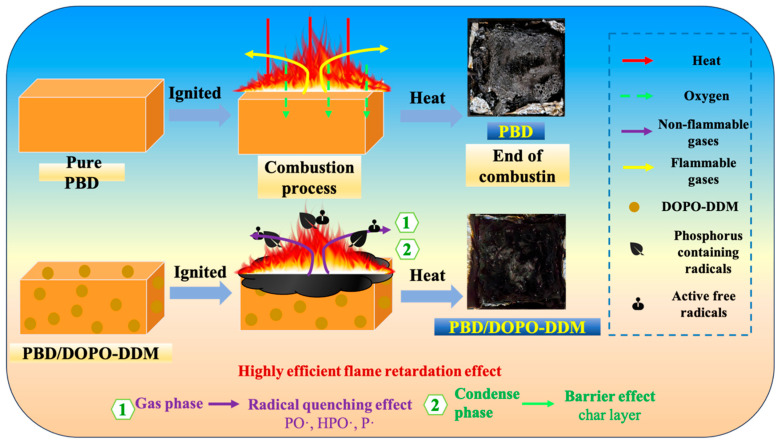
Schematic illustration of the proposed flame-retardant mechanism of Bis-DOPO Schiff-base in PBD (dominant gas-phase inhibition with an auxiliary condensed-phase surface-residue barrier).

Figure 7 summarizes the flame-retardant process of this system. TG-DTG (air, 800 °C) shows that the end-point carbon residue is only 2.3–3.5%. This indicates that the condensed phase effect is not “high residual carbon yield” or “dominated by thick carbon layers”. This system is more in line with the mechanism of “gas phase inhibition as the main and condensed phase as the auxiliary”. Pure PBD rapidly decomposes when heated. It will release a large amount of flammable volatile substances. It is difficult to form a continuous residual layer on the surface because combustion mainly occurs in the gas phase. Heat and oxygen can easily enter the interior of the material. Therefore, the heat release is higher. After the addition of Bis-DOPO Schiff-base, the flame retardant generates phosphorus-containing active species at high temperatures. They can capture free radicals such as H·/OH·, thereby weakening the flame chain reaction and lowering the combustion intensity. The rate of heat release also decreases. This gas-phase radical quenching is a typical effect of DOPO-like systems. Flame retardants can also form phosphate/phosphate ester structures in the condensed phase. They promote surface dehydrogenation and aromatization. They also promote surface carbonization. Phosphorus will be enriched in the residual layer. A more continuous and denser residual layer will form on the surface. This residual layer is usually thin. It can impede the transfer of heat, oxygen, and flammable gases during the combustion process. It provides an auxiliary shielding effect. However, under the high temperature of the air, the residual layer will still be continuously oxidized. It will gradually be consumed. Therefore, the final carbon residue of TG remains relatively low. The secondary weight loss in the high-temperature section of DTG also supports this point.

**Figure 8:**
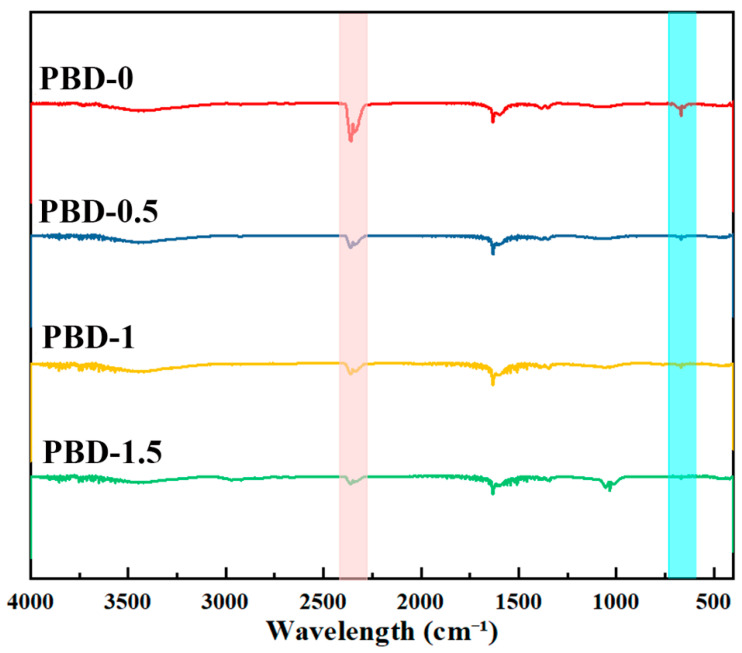
FTIR analysis of the residual char after cone calorimetry testing.

Figure 8 shows the FTIR spectrum of the residual carbon after cone calorimetry. After the addition of Bis-DOPO Schiff-base, the absorption in the 1300–900 cm^−1^ region gradually increased. This area mainly corresponds to P–O-related stretching vibrations, such as P=O, P–O–C/P–O–Ar. This indicates that when the content of flame retardants increases, more phosphorus–oxygen structures remain in the condensed phase residual carbon. This trend is consistent with the enhancement of the P signal in EDS and also with the densification of the residual carbon morphology. It is indicated that the flame retardant partially transforms into a phosphate-like solid phase at high temperatures and participates in the formation and stabilization of residual carbon. It should be noted that PBD-1 did not show clear and sharp characteristic peaks in the region of 1300–900 cm^−1^. This area is characterized by weaker broadband. This is more common in the carbon residue spectrum. The reason is that phosphate structures often present an amorphous or networked form, and the vibration peaks tend to broaden easily. Meanwhile, this area will also superimpose the wide peaks of carbonaceous residual carbon (such as C–O/C–C-related vibrations). Therefore, although the peak shape of PBD-1 is not obvious, it does not mean that the phosphorus-containing structure is lacking. Combined with its dense and complete residual carbon morphology, it can be considered that phosphorus species are fixed in the carbon layer in a more uniform manner.

The identifiable peak in the 1300–900 cm^−1^ region becomes more pronounced when the addition amount is increased to PBD-1.5. This usually means that the proportion of phosphorus-oxygen structures further increases. It may also indicate that the increase in locally enriched “phosphate-like phases” makes P–O absorption easier to distinguish from the background. But at the same time, the uniformity of the residual carbon in PBD-1.5 deteriorates, and local defects increase. Excessive flame retardants may introduce microscopic inhomogeneity, weakening the continuity of the carbon layer and shielding efficiency. In addition, the absorption of aliphatic C–H was weakened in samples with high additional amounts. This indicates that residual carbon is more prone to aromatization and carbonization. The weak peak of 700–760 cm^−1^ can be attributed to the out-of-plane bending of aromatic C–H (or residual = C–H). It weakens with the increase in the additional amount, which also indicates that the content of C–H decreases and the degree of condensation increases. The absorption at 2300–2350 cm^−1^ can be attributed to CO_2_. This peak decreases with the increase in flame retardant. This is related to the fact that the carbon layer is denser and has fewer pores. A reduction in pores will decrease the physical adsorption of CO_2_. 

**Figure 9:**
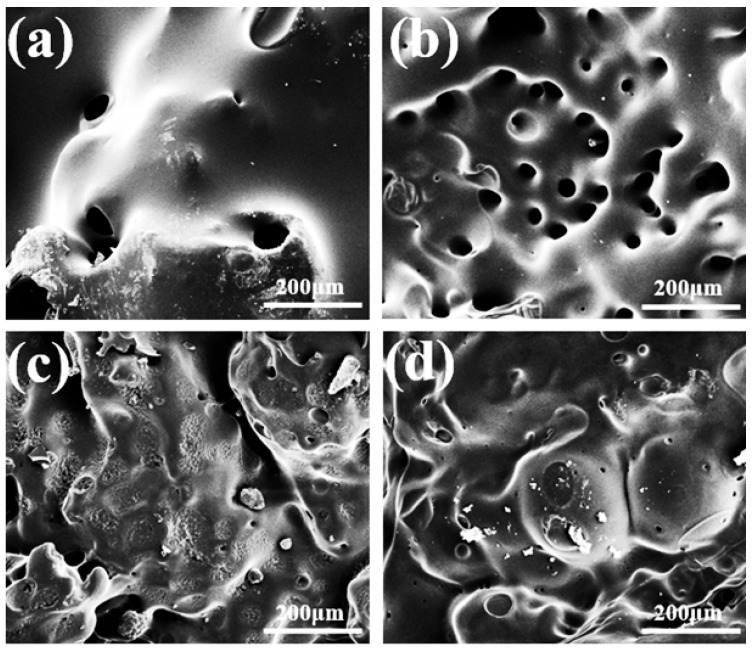
SEM images of the internal char morphology of the unmodified and flame-retardant modified Polybutadiene after combustion: (**a**) PBD-0; (**b**) PBD-0.5; (**c**) PBD-1; (**d**) PBD-1.5.

Figure 9 shows the microscopic morphology of the carbon layer inside each sample. The carbon layer pore size of PBD-0 is large. The shapes are mostly nearly circular with pore diameters ranging from tens to hundreds of micrometers. The hole walls are thin and interconnected. This indicates that the internal structure is extremely loose and almost has no effective shielding effect. After adding a small amount of flame retardant, the pores of PBD-0.5 were significantly refined. The quantity increases while the size decreases. The surface morphology is more uniform. This indicates that the carbon layer is beginning to evolve towards densification. The internal carbon layers of PBD-1 and PBD-1.5 tend to be continuous, and the number of pores further decreases. The matrix presents a relatively tight, flaky, or wrinkled structure. Fine cracks and shrinkage marks can be seen locally. This indicates that the phosphorus-rich carbon layer with certain integrity can be formed at a relatively high content of flame retardants. This path is consistent with the changes in pHRR and THR in the cone calorimetry experiment. The denser the carbon layer is, the more obviously the heat release is suppressed. It is indicated that Bis-DOPO Schiff-base promotes the formation of a stable carbon layer through catalytic dehydration and aromatization at high temperatures. This carbon layer plays a crucial role in suppressing the release of flammable volatile substances and enhancing the overall flame-retardant performance.

## 4. Conclusions

The flame-retardant performance of PBD was significantly improved by introducing Bis-DOPO Schiff-base. Flame retardants promote the formation of phosphorus-rich carbon layers at high temperatures, effectively reducing the heat release intensity and the escape rate of flammable gases during the combustion process of materials. The material achieved the best comprehensive performance at a phosphorus content of 1 wt%, reaching UL-94 V-1 grade, with a LOI of 27%, while pHRR and THR decreased by 16.1% and 19.6%, respectively. This ratio can form a carbon layer with a relatively complete structure. It provides a strong barrier effect against heat and volatile substances in the later stage of combustion. This significantly suppressed the spread of flames. The carbon layer maintains a relatively high residual amount when the content of flame retardants continues to increase. However, its structural uniformity decreases, and shrinkage and discontinuities occur locally. This leads to the flame-retardant efficiency not being further improved. Based on the overall balance of flame retardancy, thermal stability, and mechanical performance, 1 wt% is identified as the optimal loading for this system. Future work can be advanced in directions such as improving the dispersed state, optimizing the interface structure, or introducing a synergistic flame-retardant system to reduce the amount of smoke generated and enhance the structural stability of the material.

## Figures and Tables

**Table 1:** Flame-retardant PBD formulations.

Style	P (wt%)	Bis-DOPO Schiff-Base (g)	Bis-DOPO Schiff-Base (wt%)
PBD-0.5	0.5%	6.23	6.7
PBD-1	1.0%	12.46	13.5
PBD-1.5	1.5%	18.69	20.2

**Table 2:** TGA Data.

Sample	Initial Decomposition Temperature (T_5%_, °C)	Temperature at Maximum Mass Loss Rate (T*_max_*, °C)	Maximum Mass Loss Rate (%·min^−1^)	Residue at 800 °C (wt%)
PBD-0	260.42	350.0	6.8	0.4
PBD-0.5	253.075	342.5	6.5	1.6
PBD-1	246.484	333.8	6.9	2.3
PBD-1.5	243.961	322.6	7.4	3.5

## Data Availability

The original contributions presented in this study are included in the article. Further inquiries can be directed to the corresponding authors.

## Supplementary Materials

The following supporting information can be downloaded at https://www.mdpi.com/article/10.3390/polym18010127/s1, Scheme S1: The synthesis mechanism diagram of Bis-DOPO Schiff-base structure; Figure S1: Comparison of infrared spectra of Bis-DOPO Schiff-base (D-bp) and its intermediate Schiff-base; Figure S2: XPS full spectrum (a) and detailed spectrum analysis of intermediate Schiff-base (b) and Bis-DOPO Schiff-base (D-bp) (c).
