# Supplementary material for: A Phosphorus–Nitrogen Synergistic Flame Retardant for Enhanced Fire Safety of Polybutadiene
Source: Polymers (Basel). 2025 Dec 31;18(1):127. doi: 10.3390/polym18010127 (PMC12787758; doi:10.3390/polym18010127)
Supplement: Supplementary file 1 [file polymers-18-00127-s001.zip › polymers-4052017-supplementary.pdf]

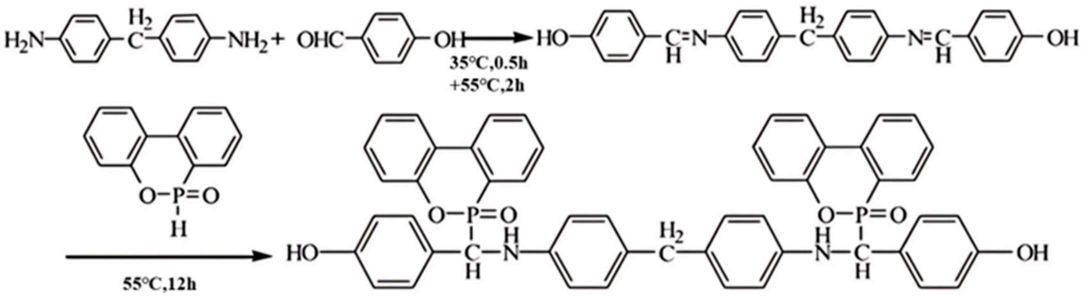

Scheme S1 The synthesis mechanism diagram of Bis-DOPO Schiff-base structure.

The FTIR spectrum (Figure S1) of the synthesized Bis-DOPO Schiff-base (D-bp) exhibits clear and characteristic absorption bands, confirming a well-defined molecular structure. Notably, the strong C=N stretching band typically observed for Schiff-base intermediates in the 1620-1640 cm^{-1} region is absent, indicating complete consumption of the imine intermediate. A distinct band at ~1274 cm^{-1} attributable to C-N stretching further supports the successful P-H addition of DOPO across the C=N bond, leading to the formation of stable P-C and C–N single-bond linkages. In addition, the presence of an N-H stretching band at ~3280 cm^{-1} and a sharp P=O stretching band at ~1236 cm^{-1} provides additional evidence for the target structure. The spectrum shows a stable baseline with no obvious impurity-related peaks, suggesting high product purity suitable for subsequent flame-retardant modification of polyurethane systems.

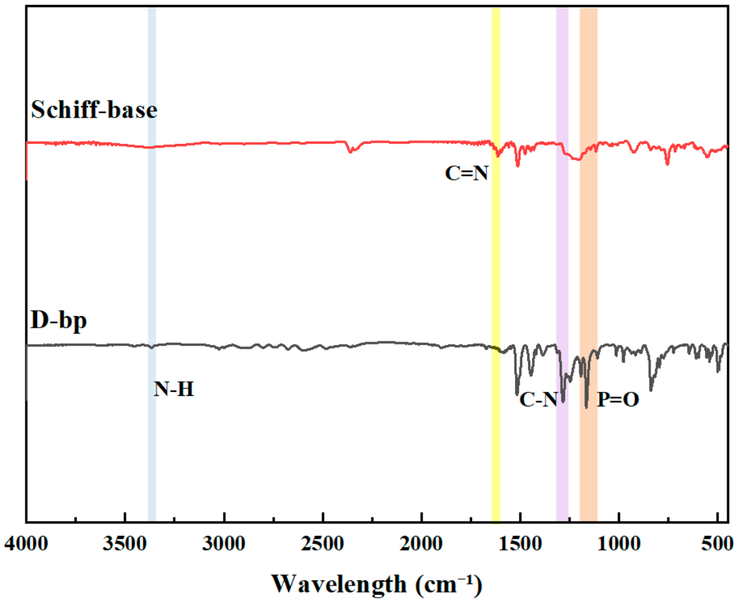

Figure S1 Comparison of infrared spectra of Bis-DOPO Schiff-base (D-bp) and its intermediate Schiff-base.

XPS analyses (Figure S2) clearly demonstrate the structural evolution from the Schiff-base intermediate to the target flame retardant (D-bp) and provide direct electronic-structure evidence for successful synthesis. In the survey spectrum, D-bp shows a distinct P2p signal at ~133 eV that is absent in the intermediate, confirming the chemical incorporation of phosphorus. High-resolution spectra further support this transformation: in the C1s region, the C=N-related component at ~286.0 eV observed for the intermediate disappears in D-bp, while a new component at ~288.5 eV emerges, attributable to carbon species influenced by the newly formed phosphorus-containing environment (e.g., carbonyl/adjacent-to-P carbon). In the N1s region, the C=N peak at ~398.8 eV vanishes and is replaced by a C-N-P component at ~399.5 eV, consistent with reduced electron density on nitrogen upon bonding to phosphorus. The O1s spectrum of D-bp exhibits peaks at ~532.0 and ~533.2 eV assigned to P=O and P-O-C, respectively, matching typical DOPO-derived P–O environments. Moreover, the P2p spectrum can be deconvoluted into contributions at ~133.8 eV ($P2p_{3/2}$, P=O) and ~132.5 eV (P-C), in full agreement with the proposed molecular structure.

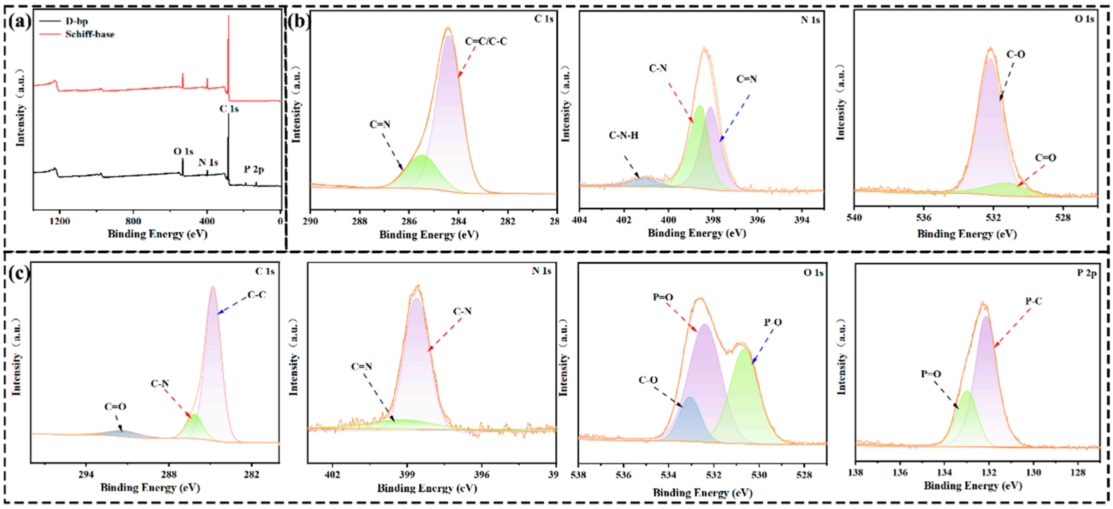

Figure S2 XPS full spectrum (a) and detailed spectrum analysis of intermediate Schiff-base (b) and Bis-DOPO Schiff-base (D-bp) (c).
